# Reduced plasma GDF10 levels are positively associated with cholesterol impairment and childhood obesity

**DOI:** 10.1038/s41598-024-51635-1

**Published:** 2024-01-20

**Authors:** Tamana R. Yousof, Aurora Mejia-Benitez, Katherine M. Morrison, Richard C. Austin

**Affiliations:** 1 Division of Nephrology, Department of Medicine, McMaster University and The Research Institute of St. Joe’s Hamilton, Hamilton, ON Canada; 2 Department of Pediatrics, Centre for Metabolism, Obesity and Diabetes Research, McMaster University, Hamilton, Canada; 3 Division of Nephrology, Department of Medicine, McMaster University and The Research Institute of St. Joe’s Hamilton, 50 Charlton Ave. E., Rm. T-3313, Hamilton, ON L8N 4A6 Canada

**Keywords:** Paediatric research, Lipids

## Abstract

Childhood obesity is a global health concern affecting over 150 million children worldwide, with projections of a rise to 206 million by 2025. Understanding the mechanisms underlying this epidemic is crucial for developing effective interventions. In this study, we investigated circulating levels of Growth Differentiation Factor 10 (GDF10), a novel regulator of adipogenesis. Previous studies report diminished circulating GDF10 levels contribute to obesity and hepatic steatosis in mice. To further understand the role of plasma GDF10 in childhood obesity, a prospective case–control study was conducted. Using an enzyme-linked immunosorbent assay, plasma GDF10 levels were measured in children aged 5–17 years of age with normal (n = 36) and increased (n = 56) body mass index (BMI). Subsequently, plasma GDF10 levels were compared to various cardio-metabolic parameters. Children with increased BMI exhibit significantly lower levels of plasma GDF10 compared to children with normal BMI (*p* < 0.05). This study not only supports previous mouse data but is the first to report that lower levels of GDF10 is associated with childhood obesity, providing an important human connection for the relevance of GDF10 in obesity. Furthermore, this study revealed a significant correlation between low plasma GDF10 levels and elevated LDL-cholesterol and total cholesterol levels dependent on BMI (95% CI,* p* < 0.05). This study supports the hypothesis that children with obesity display lower plasma levels of GDF10, which correlates with elevated cholesterol levels. These insights shed light on potential mechanisms contributing to childhood obesity and may lead to future therapeutic interventions targeting GDF10 to mitigate adverse effects of adipogenesis in cardiometabolic health.

## Introduction

The high prevalence of obesity in children presents a significant global public health concern, with numerous adverse outcomes associated with these conditions^1–9^. In Canada, nearly 30% of children aged 5–17 are classified as overweight or obese, highlighting the urgency of addressing this issue^3^. Childhood obesity often persists into adulthood, as evidenced by the fact that 77% of children with obesity become adults with obesity^10^.

Adipogenesis is the process of cell differentiation by which pre-adipocytes become mature adipocytes capable of storing lipids. Dysregulation of the adipogenic program is associated with the early onset of obesity^11^. A secreted ligand of the transforming growth factor beta superfamily termed growth differentiation factor 10 (GDF10), also known as bone morphogenic protein-3b, is expressed in all adipose depots, although higher expression is observed in pre-adipocytes than in mature adipocytes. Several reports indicate that GDF10 secretion is responsible for blocking adipogenesis^12–16^. GDF10 secretion inhibits adipogenesis by suppressing key transcriptional factors, peroxisome proliferative factor receptor gamma (PPAR**γ)** and CCAAT/enhancer-binding protein alpha^15^. GDF10’s ability to negatively regulate PPAR**γ** is also effective in reducing lipid accumulation in human hepatocytes^17^.

A recent report has shown that high-fat diet can significantly reduce fibroadipogenic progenitor-derived GDF10 transcript levels in mice, directly leading to the induction of fat infiltration in a paracrine manner^18^. We and others have also reported that mice deficient in GDF10 are prone to increased weight gain when fed a regular chow or high-fat diet^17,19^. Mice deficient in GDF10 display increased whole body abdominal adiposity and adipocyte hypertrophy, independent of changes in food consumption. Moreover, these mice develop abnormal metabolic features including impaired fasting glucose, hyperinsulinemia and dyslipidemia marked by increased circulating plasma triacylglycerol compared to wild-type controls^17^. Loss of GDF10 contributes to pathological cardiac remodeling and elevated cardiovascular risk^19^. These observations highlight the emerging potential of GDF10 as a therapeutic target due to its inverse association with obesity in vivo.

Despite this growing body of evidence, to the best of our knowledge, plasma GDF10 levels has not been correlated with BMI in children. This study not only supports previous mouse data but is the first to reinforce the notion that lower levels of GDF10 is associated with increased BMI in childhood, providing an important human connection for the relevance of GDF10 in obesity. Finally, it strongly suggests that therapies aimed at increasing plasma GDF10 levels or the pathways that are activated by GDF10 may represent a new approach for the treatment and/or management of childhood obesity.

## Materials/subjects and methods

### Study population

Study population (n = 92) of both sexes aged 5–17 years old were recruited from the Children’s Exercise and Nutrition Centre at McMaster Children’s Hospital in Hamilton, Canada and during well-child visits in a Pediatrician’s office^20^. Written and informed consent was obtained from the legal guardian with child provided assent. This study was approved by The Research Ethics Board at Hamilton Health Sciences in accordance with the Declaration of Helsinki. Demographic information (age and sex) was collected by questionnaire. All measurements were taken at a single visit the morning after a minimum fasting period of 8 h. The study visit included anthropometric measures and collection of a fasting blood sample. All study data was saved into a database as deidentified information. Considering the World Health Organization (WHO) proposed body mass index (BMI) cut-off points for adolescent populations, normal BMI is defined as BMI-for-age <  + 1SD, overweight as BMI-for-age >  + 1SD, and obesity as BMI-for-age >  + 2SD. Thus, in this study we categorized the children as normal BMI defined as BMI-for-age <  + 1SD (n = 36) and increased BMI defined as children with a BMI-for-age >  + 1SD above the mean (n = 56; 12 participants with BMI-for-age >  + 1SD and 44 with BMI-for-age >  + 2SD)^21^. The study excluded children under 5 years old or over 17 years old. The study excluded those receiving pharmacological treatment for obesity or obesity related complications based on the potential impact of health conditions and treatments that may influence food uptake among participants^22^.

### Assessments

Anthropometric parameters were measured as previously described^20^. Briefly, height was measured using a Harpenden Stadiometre (London, UK). Weight was measured using a Tanita electronic scale. BMI (kg/m^2^) and BMI-*Z* score (World Health Organization) were calculated using NUTSTAT, a component of the EpiInfo program. Waist circumference was measured half-way between the iliac crest and lower rib^23^.

Metabolic parameters were measured as previously described^20^. Fasting glucose (minimum 8 h), total cholesterol, high density lipoprotein (HDL)-cholesterol, and triglyceride levels were assessed using the Roche analyzer as previously reported^20^. Low density lipoprotein (LDL)-cholesterol was calculated according to the Friedewald formula as previously reported^20^.

### Human GDF10 measurement

Growth Differentiation Factor 10 ELISA Kit (Elabscience, Wuhan, China) was used to measure circulating GDF10 levels from plasma samples that had been stored in a – 80 °C freezer since collection and were limited to one freeze–thaw cycle as per the manufacturer’s protocol. The optimal detection range for GDF10 is 31.25–2000 pg/mL (sensitivity as low as 18.75 pg/mL). The intra- and inter-assay variations were less than 5%.

### Statistics

GraphPad software was used to perform all statistical analyses. *p* < 0.05 is considered significant for all analyses.

### Descriptive statistics of the population

Anthropometric and metabolic data were tested for normal distribution using skewness and kurtosis. Categorical traits are expressed as percentages (%) in Table 1. Comparisons of categorical traits were made between groups using the Chi-square test for independence and effect size was measured by odd’s ratio (Supplementary Table 1). Two-way ANOVA (Tukey’s multiple comparison) was performed to compare male and female age between the normal BMI and increased BMI group (Supplementary Fig. 1A). Comparison of continuous variables between the normal and increased BMI group were performed with a student’s independent T-test (two-tailed) (Table 1). Continuous variables include age (years), BMI (kg/m^2^), BMI-Z, waist to hip ratio, standardized waist to hip ratio, baseline weight, low density lipoprotein (LDL) cholesterol (mmol/L), high density lipoprotein (HDL) cholesterol (mmol/L), total cholesterol (mmol/L), triglycerides (mmol/L) and fasting glucose (mmol/L). Data is expressed as mean ± standard deviation for continuous traits.

**Table 1:** Baseline clinical and biochemical characteristics of the study subjects.

Characteristics	Total N = 92	Normal BMI N = 36	Increased BMI N = 56	*p*-value
Sociodemographic				
Female, N (%)	35 (38)	14 (38.8)	21 (37.5)	
Age (years)	11.06 ± 2.88	11.81 ± 3.10	10.57 ± 2.64	
Anthropometric measure				
BMI (kg/m^2^)	23.62 ± 7.09	17.57 ± 2.87	27.57 ± 6.17	< 0.001
Height (m)	1.47 ± 0.16	1.48 ± 0.19	1.47 ± 0.13	0.085
Waist (cm)	75.56 ± 16.91	60.64 ± 7.71	83.89 ± 14.77	< 0.001
Hip (cm)	87.19 ± 15.71	74.48 ± 12.06	93.03 ± 13.66	< 0.001
Baseline weight (kg)	52.74 ± 21.34	40.05 ± 15.56	61.05 ± 20.5	< 0.001
BMI Z-score (WHO)	1.66 ± 1.98	-0.29 ± 0.87	2.95 ± 1.36	< 0.001
Metabolic profile				
LDL-cholesterol (mmol/L)	2.28 ± 0.67	1.97 ± 0.45	2.48 ± 0.71	< 0.001
HDL-cholesterol (mmol/L)	1.40 ± 0.30	1.49 ± 0.32	1.35 ± 0.27	0.04
Total cholesterol (mmol/L)	4.20 ± 0.78	3.86 ± 0.59	4.42 ± 0.82	< 0.001
Triglycerides (mmol/L)	1.10 ± 0.77	0.87 ± 0.53	1.24 ± 0.86	0.02
Fasting glucose (mmol/L)	4.76 ± 0.45	4.77 ± 0.55	4.75 ± 0.38	0.84

Data expressed as means ± standard deviation. An independent (2-tailed) Student’s T-Test was used to compare continuous variables between normal and increased BMI groups; *P* < 0.05 is considered statistically significant.

BMI, body mass index; HDL-cholesterol, high-density lipoprotein cholesterol; LDL-cholesterol, low-density lipoprotein cholesterol.

### Investigating the association of plasma GDF10 levels with baseline characteristics

Two-way ANOVA (Tukey’s multiple comparison) was performed to compare male and female plasma GDF10 levels between the normal BMI and increased BMI group (Supplementary Fig. 1B).

The correlation coefficients and probability values in Table 2 were calculated with Pearson product moment. A Pearson’s correlation heat map of continuous anthropometric and metabolic variables is presented in Supplementary Fig. 2A. Correlation of plasma GDF10 levels compared to LDL- and total cholesterol are shown as scatterplots in Supplementary Fig. 2B.

**Table 2:** Correlation of plasma GDF10 levels with characteristics of the study subjects.

Characteristics	GDF10 (pg/ml)				
	Pearson correlation	*p*-value	N	95% CI	
				Lower	Upper
Sociodemographic					
Age	0.100	0.344	91	− 0.1079	0.3000
Anthropometric measure					
BMI (kg/m^2^)	− 0.166	0.115	91	− 0.3599	0.04103
Height (m)	0.059	0.581	91	− 0.1491	0.2614
Waist (cm)	− 0.082	0.467	81	− 0.2950	0.1389
Hip (cm)	− 0.098	0.408	73	− 0.3212	0.1347
Baseline weight (kg)	− 0.094	0.375	91	− 0.2944	0.1140
BMI Z-score (WHO)	− 0.184	0.081	91	− 0.3755	0.02307
Metabolic profile					
LDL-cholesterol (mmol/L)	− 0.227^a^	0.031	91	− 0.4133	− 0.02175
HDL-cholesterol (mmol/L)	0.069	0.515	92	− 0.1381	0.2697
Total cholesterol (mmol/L)	− 0.232^a^	0.026	92	− 0.4172	− 0.02875
Triglycerides (mmol/L)	− 0.154	0.142	92	− 0.3480	0.05234
Fasting glucose (mmol/L)	− 0.047	0.653	92	− 0.2499	0.1589

CI, confidence interval; BMI, body mass index; HDL-cholesterol, high-density lipoprotein cholesterol; LDL-cholesterol, low-density lipoprotein cholesterol.

^a^Correlation is significant at the 0.05 level (2-tailed).

Multiple linear regression (least square estimates) was used to measure the association between plasma GDF10 levels (dependent variable) and the categorical trait for BMI classification (0-normal and 1-increased BMI) adjusted for age and sex (1-male, 2-female) (Table 3). Multiple linear regression (least square estimates) was used to test the association between GDF10 (dependent variable) with LDL-cholesterol and total cholesterol adjusted for age and sex, independent of BMI (Table 3). Anthropometric markers such as BMI, BMI-Z, height, weight, and waist-to-hip ratio demonstrate multicollinearity. Similarly, metabolic markers such as triglycerides, LDL-, HDL- and total cholesterol demonstrate multicollinearity.

**Table 3:** Multiple linear regression analyses of plasma GDF10 levels and characteristics of the study subjects.

Characteristics	GDF10 (pg/ml)				
	Parameter estimate ± S.E.	*p*-value	F	95% CI	
				Lower	Upper
BMI category	− 25.53 ± 9.485^a^	< 0.01	7.247	− 44.39	− 6.682
Age	0.5273 ± 1.620	> 0.05	0.1059	− 2.693	3.748
Sex	18.10 ± 9.327	> 0.05	3.766	− 0.4390	3.748
Age- and sex-adjusted					
LDL-cholesterol	− 14.71 ± 7.081^a^	< 0.05	4.316	− 28.79	− 0.6341
Total cholesterol	− 12.17 ± 6.016^a^	< 0.05	4.095	− 24.13	− 0.2159
BMI-adjusted					
LDL-cholesterol	− 11.10 ± 7.866	> 0.05	1.991	− 26.74	4.540
Total cholesterol	− 9.932 ± 6.554	> 0.05	2.297	− 22.96	3.096

Data expressed as means ± standard error (S.E.).

^a^Association is significant at the indicated *p*-value. CI, confidence interval. BMI category: 0—normal, 1—increased. Sex: 1—male, 2—female.

**Figure 1:**
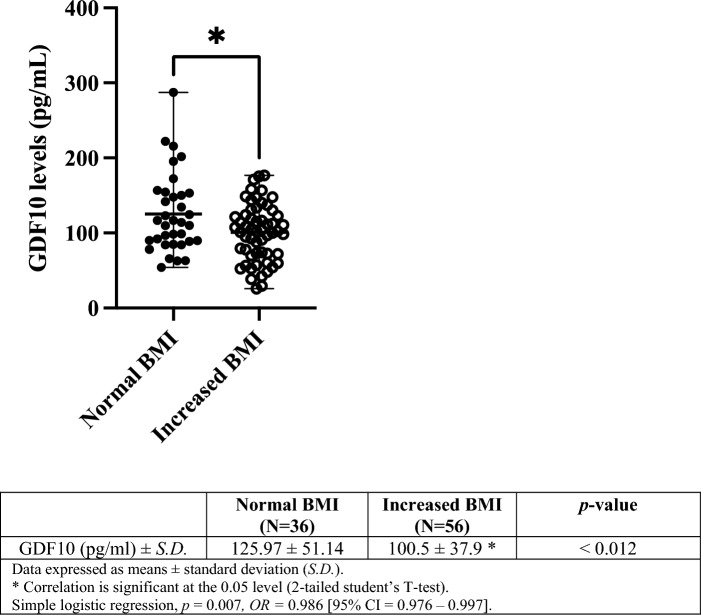
Range of plasma GDF10 levels measured in children categorized with normal BMI compared to increased BMI. Data are represented as a scatterplot of means with range.

An independent student's T-test (two-tailed) was used to test the statistical significance of plasma GDF10 levels between normal BMI and increased BMI groups (Fig. 1). A simple logistic regression was used to calculate the odd’s ratio between plasma GDF10 levels and BMI groups (normal compared to increased BMI).

## Results

### Baseline characteristics of the study subjects

Table 1 presents the baseline characteristics of the study participants, comparing children with a normal BMI (n = 36) to those with an increased BMI (n = 56). No differences in sex distribution between the two groups were identified (Supplementary Table 1). Moreover, the ages of male and female participants did not significantly differ between normal BMI and increased BMI groups (Supplementary Fig. 1). Thus, age and sex were comparable between groups.

Consistent with group assignment, children in the increased BMI group exhibited significantly higher values for BMI, waist-to-hip ratio, weight, and BMI Z-score. However, there were no significant differences in height between the study groups. Children in the increased BMI group also had higher circulating levels of triglycerides, total cholesterol, and LDL-cholesterol and lower levels of HDL-cholesterol (Table 1). These findings are consistent with the expectation that children with obesity exhibit an at-risk lipid profile^24–27^. The levels of fasting glucose were within normal range and did not differ significantly between the normal and increased BMI groups.

Plasma GDF10 levels were similar in males and females in both the normal BMI and increased BMI groups suggesting limited effect of sex (Supp Fig. 1B). Table 2 provides correlations between plasma GDF10 levels and various anthropometric and metabolic parameters. Although there was a trend towards a negative correlation between plasma GDF10 levels and several anthropometric measures including BMI, BMI Z-score (WHO), waist circumference, hip circumference, and body weight, these correlations were not statistically significant (Table 2).

### Plasma GDF10 levels are significantly reduced in children with increased BMI and inversely associated with LDL- and total cholesterol in a BMI-dependent manner

Plasma GDF10 levels were inversely correlated with total cholesterol (correlation coefficient: − 0.232, *p* = 0.026) and LDL-cholesterol (correlation coefficient: − 0.227, *p* = 0.03) (Table 2) in univariate analysis (Supp. Fig. 2). Multiple linear regression analyses indicate that GDF10 is negatively associated with LDL- and total cholesterol when adjusted for age and sex, however, this significant association is dependent on BMI (Table 3).

Plasma GDF10 levels are significantly reduced in children with increased BMI compared to normal BMI (*p* < 0.012) (Fig. 1). Multiple linear regression tested the relationship between plasma GDF10 levels as a continuous trait associated with BMI classification (Table 3). BMI classification is a significant predictor of plasma GDF10 levels when adjusted for age and sex (unadjusted analyses also demonstrate a significant association), the higher classification assigned to increased BMI negatively correlates with plasma GDF10 levels (*p* < 0.01) (Table 3). Cumulatively, this data suggests a relationship between lower plasma GDF10 levels and adiposity regulation.

## Discussion

### Lower plasma GDF10 levels in youth with increased BMI

Accumulating evidence demonstrates the ability of GDF10 to negatively regulate adiposity by suppressing key adipogenic transcription factors in rodent models^15–17,28^. Understanding the mechanistic role of GDF10 secretion in obesity is of paramount importance as it offers valuable insights into the underlying processes involved in adiposity regulation. This study reports significantly reduced plasma GDF10 levels in children with increased BMI compared to those with normal BMI. These findings suggest that lower GDF10 secretion may play a role in the development and progression of obesity or that plasma levels of GDF10 are reduced in the context of obesity.

This study is the first to report the inverse correlation between plasma GDF10 levels and LDL-cholesterol and total cholesterol levels in children. The relationship between plasma GDF10 levels and cholesterol metabolism is dependent on BMI. These findings are consistent with the reported loss of GDF10 resulting in dyslipidemia and hypercholesterolemia in mouse models of cardiovascular disease and obesity^19^. Following a high-fat meal, single-nucleotide polymorphisms of GDF10 was linked to blood pressure loci and shown to impact lipid metabolism^29,30^. GDF10’s protective role against dyslipidemia and hypercholesterolemia may be attributed to its inhibitory effect on PPAR*γ* as previously shown^15–17^. Further research is warranted to elucidate the causal relationship between GDF10-mediated inhibition of PPAR*γ* and cholesterol metabolism independent of obesity*.*

While the study provides initial insights into the association between plasma GDF10 levels, childhood obesity, and circulating lipids, it is important to acknowledge study limitations. These limitations include small sample size, limited age range, and a higher proportion of males than females. These factors restrict generalizability and increase the likelihood of random variations and reduced statistical power, which may limit the ability to draw definitive conclusions. The study focused on a specific age range, which only include children and youth, without participants from other age groups. Consequently, the findings may not fully represent the broader population, and the observed associations may differ in different age groups, such as adults or older individuals. Future studies should aim to include a wider age range in a larger and more diverse cohort, to validate and expand upon the association between plasma GDF10 levels and obesity, as measured by BMI.

## Conclusion

To our knowledge, this is the first study providing compelling evidence that children with high BMI exhibit lower plasma levels of GDF10, compared to those with normal BMI. Specifically, the observed BMI-dependent inverse association between plasma GDF10 levels and LDL-cholesterol and total cholesterol levels highlights the potential role of GDF10 in regulating lipid metabolism within the context of childhood obesity. Further research should examine the intracellular processing and secretion of GDF10 specifically in children, recognizing that these mechanisms may differ from those observed in adults. A comprehensive understanding of the mechanisms underlying GDF10 secretion and its impact on obesity will potentially identify novel targets for interventions aimed at mitigating the adverse effects of childhood obesity. Developing interventions that specifically modulate GDF10 expression or activity could improve BMI reduction, cholesterol management, and overall metabolic health in affected children.

However, further research is necessary to elucidate the mechanisms by which GDF10 regulates metabolism and determine the most effective strategies for targeting this protein. Clinical trials and preclinical studies are warranted to evaluate the safety, efficacy, and feasibility of GDF10-based therapies in children with high BMI and high cholesterol.

### Supplementary Information

Supplementary Information.

## Data Availability

All data generated or analysed during this study are included in this published article and its supplementary information files.

## Abbreviations

BMI: Body mass index
GDF10: Growth differentiation factor 10
HDL: High-density lipoprotein
LDL: Low-density lipoprotein
SMAD: Mothers against decapentaplegic
