# Supplementary material for: Reduced plasma GDF10 levels are positively associated with cholesterol impairment and childhood obesity
Source: Sci Rep. 2024 Jan 20;14:1805. doi: 10.1038/s41598-024-51635-1 (PMC10799949; doi:10.1038/s41598-024-51635-1)
Supplement: Supplementary file 1 — Supplementary Information. [file 41598_2024_51635_MOESM1_ESM.docx]

**Supplementary Information.**

**Supplementary Table 1.** Frequency of observed and expected number of male and female children with normal or increased BMI.

| **Frequency, N**  **Observed (Expected)** | **Normal BMI** | **Increased BMI** | Total |
| --- | --- | --- | --- |
| Male | 22 (22.3) | 35 (34.7) | 57 |
| Female | 14 (13.7) | 21 (21.3) | 35 |
| Total | 36 | 56 | 92 |

Χ^2^ (1, N = 92) = 0.0179, p = 0.89, OR = 1.06

**Supplementary Figure 1A.** Scatterplot of male and female participant’s age in normal and increased BMI groups.

Two-way ANOVA (Tukey’s multiple comparisons), Normal BMI Male vs Female, p > 0.05; Increased BMI Male vs Female, p > 0.05.

**Supplementary Figure 1B.** Scatterplot of male and female participant’s plasma GDF10 levels in normal and increased BMI groups.

Two-way ANOVA (Tukey’s multiple comparisons), Normal BMI Male vs Female, p > 0.05; Increased BMI Male vs Female, p > 0.05.

**Supplementary Figure 2A.** Pearson’s correlation heat map of continuous anthropometric and metabolic variables.

**Supplementary Figure 2B.** Plasma GDF10 levels correlate negatively with (*left*) LDL-cholesterol (N=91, *p*= 0.031) and (*right*) total cholesterol (N=92, *p*= 0.026).

Abbreviations: LDL- cholesterol, low-density lipoprotein cholesterol.
